# Case of Lumbar Abscess in a Horse

**Published:** 1829-01-01

**Authors:** 


### Lumbar Abscess in a Horse.

When man, with that instinctive impulse towards improvement, which at present has the nickname of the March of Intellect, forms social connexions, and advances in civilization, he drags in his *wake* the inferior animals, employs them for his ends, and gives them in return an artificial subsistence, with many of the ills his habits or his vices have entailed upon himself. Of all the animals that man thus uses, or, perhaps abuses, the horse is the most valuable; in other words, works the hardest for the food he gets. The diseases of horses are at present nearly as numerous as those of their masters, and the "Veterinarians" of the day are just as philosophic in their manipulations and manœuvres, and as scientific in their treatment and descriptions of *equine* disorders as any of the faculty in any complaint in the nosology. For our own parts, it would not surprise us in the least to witness a goodly octavo issue from the press, on dyspepsia in horses, or a paper in some veterinarian transactions, on hysterical affections in mares.* We were led to these reflections, from observing in the Midland Reporter, a case of lumbar abscess in a horse, and confess we were not a little amused at observing the professional style of narrating it. We had read it nearly through before we discovered that the patient was a horse, and not a christian. As a matter of curiosity we give the *case*, which is short, in the words of the author, who appears an intelligent Veterinary surgeon.

"April 4, 1826, a bay gelding, 6 years old, then the property of Mr. W. residing near this city, was sent for my inspection. I soon discovered that he was lame, but the proximate cause of the lameness was not so obvious. At times he went very sound and well, and then, in an instant, became incapable of moving the near hind Leg, the muscles of which seemed to be simultaneously and rigidly affected with Spasm. This attack continued for several seconds, after which, he could go on as well as usual for a short distance, (perhaps 80 or 100 yards,) when the same thing almost invariably recurred. Pressure on the Loins produced the same symptoms, which subsided as quickly as in the former instance. He did not, apparently, suffer much pain when in a quiescent state, the Pulse being but little accelerated. His Bowels were regular, and he voided his Urine in sufficient quantity, and without difficulty. I could not recognise the slightest defect in the action of the off hind Leg, nor could I, upon the most accurate examination of the near one, discover any thing to account for the lameness.

"The information I received from the person who brought him to my stables, was, that he had been ridden with the fox-hounds a few days before, and was first found to fail immediately after leaping a high gate at the end of a run.

"I treated this case by copious bloodletting, administering an active Cathartic, blistering extensively over the lumbar region, light diet, warm clothing, and perfect quietude. The Cathartic and Blister were occasionally repeated. In the beginning of May, he seemed a little better; a stimulating Adhesive Plaster was then laid upon the blistered surface, and he was sent to grass. He somewhat improved in the course of the Summer, until the latter end of August, when febrile disturbance shewed itself in the system, and the lameness very much increased. At this time I entertained little hopes of his recovery. He was become very lean and emaciated, and continued to lose flesh and strength daily. On visiting him on the 3rd of September, I perceived a small Tumour on the off side of the Anus, from which a little Pus oozed out. I immediately opened it, and a very considerable quantity of healthy looking matter escaped. I enlarged the Orifice with a bistoury, so that the Pus could have free exit, and afterwards examined the Abscess with a probe,

---

* Qy. *Mayor-esses?* Prs. Dev.

which I passed (evidently from the length and direction of it,) into the substance of the Psoæ Muscles. The Sinus crossed the Rectum obliquely, the Abscess (as far as I could judge from probing the wound,) being situated on the near side of the Spine. No dressing of any kind was applied, and as the weather was mild he was left at grass. From this time I may date my Patient's recovery; his lameness soon got better, the discharge gradually diminished, and he gained flesh rapidly. By the latter end of October, the Sinus was nearly filled with healthy granulations, and the discharge was very trifling. Before Christmas, the Wound was quite healed, and he was sold. I saw no more of him until November, 1827, when I met him by accident, one day, in the streets, looking very healthy, and in good condition. He was afterwards purchased by a very celebrated sportsman in this county, who told me he rode him several times with the fox-hounds last season, and was carried quite to his satisfaction, and that, as a hack, he found him very sound and effective.

G. CORBETT, Veterinary Surgeon."

If the above was really a case of psoas abscess, (and we know much too little of the anatomy or diseases of horses to decide,) the issue was different from that which results in the human subject.

---

